# Essential Oils in Nellore Beef Cattle: In Vivo Impact on Rumen Emissions

**DOI:** 10.3390/ani14111664

**Published:** 2024-06-02

**Authors:** Gabriela Benetel, Gisele Maria Fagundes, Paulo de Méo-Filho, Thaysa dos Santos Silva, Katiéli Caroline Welter, Flávia Alves Melo, Annelise Aila Gomes Lobo, Rosa Toyoko Shiraishi Frighetto, Alexandre Berndt, James Pierre Muir, Ives Cláudio da Silva Bueno

**Affiliations:** 1 Department of Animal Science, Universidade de São Paulo—USP, Av. Duque de Caxias Norte, 225, Pirassununga 13635-900, SP, Brazil; gabriela.benetel@usp.br (G.B.); thaysamariana@hotmail.com (T.d.S.S.); katieliwelter@yahoo.com.br (K.C.W.); flaviamelo.zoo@gmail.br (F.A.M.); anneliselobo@usp.br (A.A.G.L.); ivesbueno@usp.br (I.C.d.S.B.); 2 Department of Animal Science, Universidade Federal de Roraima—UFRR, BR 174, Km 12, Boa Vista 69300-000, RR, Brazil; 3 Department of Animal Science, University of California—UC Davis, 1 Shields Ave, Davis, CA 95616, USA; paulo.filho@usp.br; 4 Research and Development, Embrapa Meio Ambiente, Rod SP-340, Km 127, Jaguariúna 13820-000, SP, Brazil; rosa@cnpma.embrapa.br; 5 Research and Development, Embrapa Pecuária Sudeste, Rod Washington Luiz, Km 23, São Carlos 13560-970, SP, Brazil; alexandre.berndt@embrapa.br; 6 Texas A&M AgriLife Research, 1229 North U.S. Hwy 281, Stephenville, TX 76401, USA; jim.muir@ag.tamu.edu

**Keywords:** greenhouse gases, oregano, ruminants, SF6 tracer technique, thyme

## Abstract

**Simple Summary:**

The impact of livestock on climate change has prompted animal nutrition researchers to reassess ruminant feeding strategies that minimize negative repercussions on the ecosystem. Essential oils (EOs), derived from natural plant extracts, have emerged as promising substances capable of inhibiting rumen methanogenesis. This study elucidated the effects of oregano and white thyme EOs on in vivo rumen methane emissions and rumen parameters in Nellore beef cattle. In contrast to results obtained through in vitro methods, our in vivo results indicated that oregano and white thyme EOs included in the diet at 3 mL/kg dry matter were not effective in reducing rumen methane emissions.

**Abstract:**

Essential oils (EOs), as rumen additives, decreased CH_4_ emissions in in vitro trials but results from in vivo studies are still limited. We investigated the effects of *Origanum vulgare* (OEO) and *Thymus vulgaris* (TEO) EOs on in vivo methane emissions from Nellore beef cattle. Six adult rumen-cannulated Nellore cattle were used in a double 3 × 3 Latin square design. Treatments consisted of three diets containing either 3 mL OEO per kg of concentrate, 3 mL TEO/kg of concentrate, or no EO addition. The experimental period consisted of three 21 d feeding periods and methane production was measured using the sulfur hexafluoride (SF6) technique from Day 16 to Day 21 of each feeding period. Intake, total apparent digestibility (dry matter as well as neutral and acid detergent fiber), and rumen parameters (pH, ammoniacal nitrogen concentration, and short-chain fatty acids) were also evaluated. The EOs did not decrease CH_4_ emissions and had no effect on rumen parameters.

## 1. Introduction

Beef cattle grazing on extensive tropical pastures are significant contributors to fermentative methane production, exacerbating climate change [1]. The use of ionophores as growth promoters in livestock can lead to the accumulation of residues in milk and meat, resulting in environmental contamination and promoting antibiotic resistance in humans [2]. Consequently, there is an urgent need for natural and safe alternatives to ionophore antibiotics that can reduce ruminal methane emissions. Recent studies have highlighted the potential of essential oils (EOs) to modify ruminal fermentation [3,4], alter rumen microbial populations [5], and suppress methanogenesis [6].

EOs are complex mixtures of compounds with diverse functions and mechanisms of action. Their hydrophobic nature enables them to interact with the lipids in bacterial cell membranes and mitochondria, altering membrane structure and increasing permeability. This disruption causes leakage of ions and other cytoplasmic contents, thereby exerting antimicrobial effects [7]. The predominant compound and its concentration in each plant extract determine its specific mode of action and function [8]. Although a wide variety of EOs are available, there is limited literature on their effects in cattle feed and the recommended dosages for animal diets.

Busquet et al. [9] demonstrated, in vitro, that garlic oil and diallyl disulfide (at 300 mg/L) reduced rumen CH_4_ production by 74% and 69%, respectively, which was more effective than monensin (42% reduction). Similarly, Patra et al. [10] investigated the in vitro effects of various natural plant extracts, including clove; fennel; onion; garlic; and ginger, on methanogenesis.

However, positive in vitro results do not always translate to in vivo scenarios [11]. Essential oils may have distinct odors and flavors that affect palatability and intake in animals, and the required in vivo dosages may lead to adverse effects or be impractical. In vitro studies allow precise control over ruminal parameters such as pH, osmolarity, and retention time, which cannot be similarly manipulated in vivo. Factors like compound degradation in the rumen, microbial adaptation, passage kinetics, pH fluctuations, osmotic gradients, volatilization, and absorption through the ruminal mucosa contribute to the discrepancies between in vitro and in vivo outcomes [4].

In vitro EO dosages often exceed those needed in vivo due to the lower microbial concentrations in laboratory settings compared to natural environments. Therefore, in vivo trials are essential to thoroughly evaluate the impact of EOs on ruminal microbial fermentation. Among various EOs, *O. vulgare* and *T. vulgaris* contain active compounds such as thymol and carvacrol, which have been shown to mitigate rumen methane emissions in vitro [6]. There is a pressing need for in vivo research to provide reliable information on the use of EOs, their active principles, and recommended dosages for environmentally friendly applications [12]. Our research aimed to contribute to sustainable nutritional strategies that enhance productivity while reducing rumen methane emissions from cattle, thereby supporting global livestock sustainability. This study focused on evaluating the impact of incorporating *T. vulgaris* and *O. vulgare* as nutritional strategies to mitigate in vivo methane emissions in Nellore cattle.

## 2. Materials and Methods

### 2.1. Experiment Area

This research was conducted at the Experimental Feedlot Sector of the Animal Science Department, College of Animal Science and Food Engineering, in collaboration with the University of Sao Paulo, Embrapa Pecuária Sudeste, and Embrapa Meio Ambiente. All these institutions are situated in the State of Sao Paulo, Brazil. The study was approved by the ethics committee of the University of Sao Paulo Department of Animal Science (2416120916).

### 2.2. Diets and Experimental Setup

Six adult rumen-cannulated Nellore cattle, castrated males with a body weight of 591.7 ± 28.8 kg and a body condition score of 8, were randomly assigned to individual feeding crates in a double 3 × 3 Latin square design. The treatments comprised three diets: a control diet with no EO, a diet with 3 mL of oregano EO (OEO) per kg of concentrate on a dry matter (DM) basis, and a diet with 3 mL of thyme EO (TEO) per kg of concentrate on a dry matter (DM) basis. These concentrations were based on our previous in vitro studies [6]. Within each group (square), two steers were assigned to each of the three diets.

The trial extended over 63 days, with each feeding period lasting 21 days (14 days for adaptation and 7 days for sample collection). The experimental diets, formulated to meet NRC [13] maintenance requirements, consisted of a total mixed ration based on 70% *Zea mays* silage and 30% concentrate. The concentrate included ground *Z. mays* (20% DM concentration basis), *Glycine max* meal (6.5% DM concentration basis), soybean oil (1% DM concentration basis), common salt (0.5% DM concentration basis), and mineral vitamin nucleus (Minerthal Flexbeef^®^ Araçatuba, SP, Brazil at 2%, DM basis). Details of diet ingredients and chemical compositions are presented in Table 1. Essential oils from Ferquima^®^ (São Paulo, SP, Brazil) were added to the concentrate. The animals were fed twice daily at 8:00 a.m. and 16:00 p.m. Diet components were thoroughly mixed before being offered to the cattle.

### 2.3. Measurement and Sampling Procedures

Daily recording of diet DM intake (DMI) was conducted by weighing the total ration offered and the amount refused by the animal. Samples of diets, refusals and feces were collected from Days 15 to 19 in each experimental cycle. Fecal samples were directly obtained from the rectum of the animals using plastic gloves. Subsequently, the diet, refusals, and fecal samples were subjected to a 72 h drying period in a forced-air oven at 55 °C and ground through a 1 mm screen in a Wiley mill (Thomas Scientific, Swedesboro, NJ, USA). To estimate apparent digestibility, indigestible neutral detergent fiber (NDFi) and indigestible acid detergent fiber (ADFi) were utilized as internal markers, following methodology outlined by Cochran et al. [14]. Each pre-dried sample (feed, leftovers, and feces) underwent three repetitions and was packed in 10 × 5 cm non-woven tissue bags (TNT 100 g/m^2^) at a ratio of up to 20 mg DM per cm^2^ surface area [15]. These bags were then enclosed in Raschel-type nets with accompanying apparatus for improved handling and incubation of the samples. After 288 h, the bags were retrieved from the rumen, washed with running water, and subsequently subjected to NDF and ADF analyses using Tecnal^®^ fiber analyzer equipment (Piracicaba, SP, Brazil), each lasting for 1 h. Following this procedure, the samples were washed with hot water, followed by acetone, and finally dried in a non-ventilated oven (105 °C for 4 h). They were then placed in a desiccator and weighed, thus determining the concentrations of NDFi and ADFi.

### 2.4. Methane Measurements

Methane emissions were quantified utilizing the sulfur hexafluoride (SF6) technique, as outlined by Johnson et al. [16], adapted by Primavesi et al. [17], and refined by Berndt et al. [18]. The animals were equipped with gas collection hoists linked to PVC (polyvinyl chloride) yokes (60 mm class 20), pre-evacuated to allow 50% filling within a 24 h period. The animals underwent a 1-week acclimatization period wearing the sample equipment before the commencement of collection procedures. To initiate measurements, a permeation tube containing ultrapure SF6 was inserted into the rumen of each animal one week before the commencement of measurements in the first period. Exhaled methane collections were conducted over 24 h and repeated for five consecutive days at consistent times. Background levels of SF6 and methane were measured daily by suspending canisters within the stall. Air samples from collection canisters were analyzed for methane and SF6 concentrations at Embrapa Meio Ambiente, Jaguariúna, SP, Brazil, using a Hewlett Packard^®^ model 6890 gas chromatograph (Agilent, San Jose, CA, USA) equipped with flame ionization detectors (FID) at 280 °C. The column (0.53 mm × 30 m, 15 μm) HP-MolSiv (for SF6) included two 0.5 cm^3^ loops maintained at 80 °C attached to two six-way valves, as described by Johnson et al. [14]. The column temperature was sustained at 50 °C during analysis and heated to 150 °C for approximately 15 min for cleaning purposes. Calibration curves were established using standard gas certified by the company White Martins^®^ (Bauru, SP, Brazil) with concentrations of CH_4_ in parts per million (ppm), following the methodology of Westberg et al. [19].

The potential CH_4_ emission was expressed in different units, as follows: grams of methane per day = CH_4_ (g.day^−1^); kilograms of methane per year = CH_4_ (kg.year^−1^); grams of methane per kilogram of dry matter intake = CH_4_ (g.kg DMI^−1^); grams per kilogram of live weight = CH_4_ (g.kg BW^−1^); and grams per kilogram of metabolic weight = CH_4_ (g.kg^−1^ BW^0.75^). The percentage of gross energy lost in the form of CH_4_ (YM%) was the percentage ratio between energy lost in the form of methane and gross energy ingested.

### 2.5. Ruminal Fermentation Measurements

On the 21st day of each period, samples of rumen content were collected from the animals at various times: the days before (0) as well as 4, 8, and 12 h after the morning feeding. Rumen contents were manually obtained from three distinct points (frontal, median, and caudal portions) via a ruminal cannula and subsequently filtered through two layers of draining tissue to extract the ruminal fluid. This fluid was then utilized to assess ruminal fermentation parameters, including pH, short-chain fatty acids (SCFAs), and ammonia nitrogen (NH_3_-N). The pH was promptly determined post-extraction using a portable potentiometer (HANNA Instruments^®^ HI8424, São Paulo, SP, Brazil).

For SCFA determination, a 4 mL aliquot of the filtered liquid content was collected and promptly stored in a labeled glass bottle, sealed, and placed in a freezer at −20 °C. Subsequently, the samples underwent analysis using a gas chromatograph (GC-2014, Shimadzu^®^, Kyoto, Japan) equipped with a capillary column (Stabilwax^®^, Restek, Centre County, PA, USA) maintained at 145 °C (isothermal). The system included a split/splitless injector and dual flame ionization detectors (FID) operating at 250 °C, following the method outlined by Bueno et al. in 2020 [20]. Helium gas served as the carrier gas, with synthetic air as an oxidizer and hydrogen as a fuel. For the analysis, the material was thawed, subjected to centrifugation (14,500 g for 10 min), and then 0.8 mL of the supernatant was withdrawn. To this, 0.2 mL of formic acid (98–100% P.A. ACS) and 0.1 mL of the internal standard (2-ethyl-butyric acid 100 mM, Chemservise, West Chester, PA, USA) were added. An external standard, prepared with acetic, propionic, isobutyric, butyric, isovaleric, and valeric acids (Chemservice, USA), was employed for calibration. GCSolution^®^ software Ver. 2.32 (Shimadzu, Kyoto, Japan) was utilized for the calculations.

Ruminal samples (2 mL each) were collected and preserved in glass bottles containing 1 mL of 1 N sulfuric acid for the determination of N-NH_3_ concentrations. The bottles were then sealed, labeled, and stored in a freezer until the time of ammonia nitrogen determination. The analyses were conducted using colorimetry, following the procedure outlined by Kulasek (1972) [21] and adapted by Foldager (1977) [22].

### 2.6. Chemical Analysis

The quantification of DM (ID 930.15), mineral matter (MM) (ID 942.05), organic matter (ID 942.05), crude protein (CP) (ID 954.01), and ether extract (EE) (ID 920.39) was executed in accordance with AOAC (1998) standards. Neutral detergent fiber was determined sequentially, as per Mertens [23], involving the addition of α-amylase and considering residual ash. Acid detergent fiber (ID 973.18) determination followed the procedures outlined by AOAC [24]. The total carbohydrate (TC) was calculated using the equation: TC = OM − [CP + EE] [25]. The non-fiber carbohydrate (NFC) concentrations of the feed were estimated by the following equation: 100 − (CP% + NDF% + ash% + EE %), described by Sniffen et al. (1992) [25].

### 2.7. Statistical Analysis

The DMI data, CH_4_ emission, and rumen parameters were subjected to analysis of variance using the Statistical Analysis System program (Version 9.3, 2011) through the MIXED procedure. Treatment was considered a fixed factor, considering the period effect, animal within square effect, and square effect as random effects. Different collection moments were also considered as a factor of repeated measurements over time. The treatment effect was further dissected using Tukey’s 5% probability test. Time analysis was only conducted when the interactions between the time effect and treatment effect were found to be significant. Results were considered significant at *p* ≤ 0.05.

The statistical model used was as follows:

$$Y_{ijkl}=\mu +l_{j}+c_{k}+q_{l}+t_{i}+e_{ijkl}$$

where $Y_{ijkl}$ is the dependent variable, μ is the mean, $l_{j}$ is the random period effect j (j = 1, 2, 3), c*_k_* is the k animal random effect in each square (k = 1, 2, 3, 4, 5, 6), $q_{l}$ is the random effect of square q (q = 1, 2), $t_{i}$ is the treatment fixed effect i (i= 1, 2, 3), and e $e_{ijkl}$ is the random error ijkl. 

This followed the statistical model used for the data on rumen parameters:

$$Y_{ijklt}=\mu +l_{j}+c_{k}+q_{l}+a_{i}+b_{t}{+{\left(ab\right)}_{ti}+e}_{ijkl}$$

where $Y_{ijkl}$ is the dependent variable, μ is the mean, $l_{j}$ is the random period effect j (j = 1, 2, 3), c_k_ is the k animal random effect in each square (k = 1, 2, 3, 4, 5, 6), $q_{l}$ is the random effect of square q (q = 1, 2), $a_{i}$ is the i diet fixed effect (i= 1, 2, 3), $b_{t}$ is the t time factor fixed effect (t = 1, 2, 3, 4), ${\left(ab\right)}_{ti}$ is the interaction effect between diet factor i and time t, and e $e_{ijkl}$ is the random error ijkl. 

## 3. Results

### 3.1. Intake, Digestibility, and Methane Emissions

The addition of OEO or TEO to feed had no effect on DMI in cattle compared to those fed the control diet (Table 2). The digestibility of DM and nutrients was influenced by various factors, including ingredients and additives. Nevertheless, as evidenced in Table 2, there were no effects (*p* > 0.05) attributable to adding OE on the apparent digestibility of DM, NDF, or ADF. 

When assessing methane emissions (Table 3), no differences were observed between the treatment groups receiving essential oils (EOs) and those that did not receive the essential oils (*p* > 0.05).

### 3.2. Ruminal Fermentation

The mean values of ruminal pH, N-NH_3_, and short-chain fatty acid (SCFA) concentrations are presented in Table 4. As evident, there were no noteworthy effects of treatments or the interactions between treatments and time (*p* > 0.05). Differences among sampling times were observed for pH and N-NH_3_ (*p* < 0.05). At 12 h post-feeding, the lowest pH (6.25) and N-NH_3_ concentration (4 mg dL^−1^) was observed in the rumen of cattle.

## 4. Discussion

### 4.1. Intake, Digestibility, and Methane Emissions

Our investigation into the effects of oregano essential oil (OEO) and thyme essential oil (TEO) on intake, digestibility, and methane emissions in ruminants revealed several notable findings. Despite the distinctive taste and odor characteristics of EOs, which can influence feed intake [8,26], our results demonstrated no significant alterations (*p* > 0.05) in consumption-related variables with the inclusion of OEO or TEO. This is consistent with Fugita [27], who also observed no significant changes in intake with different EO blends. Conversely, Hristov et al. [28] reported reduced dry matter intake (DMI) in dairy cows fed with OEO, suggesting a degree of variability contingent upon the specific EO formulation and its administration.

The literature on the effects of TEO and OEO in forage-rich diets for cattle remains sparse. While Meyer et al. [29] reported no difference in DMI with a commercial EO product in high-grain diets, Giller et al. [30] noted a 4% increase in DMI with a blend of EOs, indicating that the impacts of EOs on intake are diet-dependent and may vary with the specific EO formulation used.

Digestibility is a pivotal factor in ruminant nutrition, influenced by dietary components and additives [31]. Our study found that EO inclusion at 3 mg/day did not affect the apparent digestibility coefficients of DM, NDF, or ADF, aligning with results from Lin et al. [32], Benchaar et al. [33] and Meyer et al. [29], who also reported no significant changes with various EO treatments. Nonetheless, studies such as those by Kurniawati et al. [34] and Metwally [35] have shown that EOs can either decrease or enhance digestibility based on the EO type and dosage, underscoring the complexity and context-dependency of EO effects.

Methane emissions are a critical environmental concern in ruminant agriculture. Despite evidence that certain EOs can reduce methane production by affecting fermentation and microbial populations [36], our study did not find significant reductions with OEO or TEO at 3 mL/kg of concentrate. This inefficacy could be attributed to the dosage being insufficient to impact methanogenic archaea populations. Previous studies by Benchaar and Greathead [37] and Cobellis et al. [38] indicate that higher EO doses are required to inhibit methanogenesis, although these can adversely affect feed digestion and productivity. Our in vitro results suggested potential methane reduction with OEO and TEO, but these effects were not replicated in vivo, consistent with findings by Olijhoek et al. [39] and Stefenoni et al. [40]. 

Furthermore, we hypothesized a potential simultaneous increase in both methane-producing and methane-utilizing microbial populations. Our revised analysis, drawing on additional literature and preliminary in vitro data, suggests that while there may be enhanced activity of methane-utilizing bacteria, the predominant effect of essential oils (EOs) appears to be the suppression of methanogen growth and activity. This suppression likely contributes to an overall reduction in methane emissions. Further research is imperative to elucidate the mechanisms of EO action and determine effective dosages that balance environmental benefits and productivity. 

### 4.2. Ruminal Fermentation

Our study did not observe significant impacts of EO treatments on ruminal fermentation characteristics, which may be attributed to the unchanged nutrient intake and digestibility. Maintaining optimal ruminal pH is crucial for microbial activity and fiber digestion [41], and the pH values in our study ranged from 6.2 to 7.1, within the recommended range for effective ruminal fermentation. This suggests that the EO doses used did not adversely affect ruminal pH or microbial populations.

Several studies [42,43,44] corroborate our findings, reporting no significant changes in ruminal pH with various EO treatments. However, the effect of EOs can be modulated by the diet’s concentrate content, as higher acidity enhances EO interaction with cell membranes [3,45]. The absence of pH changes in our study may elucidate the lack of effect on short-chain fatty acid (SCFA) concentrations, which are closely correlated with ruminal pH and play a pivotal role in energy metabolism [46].

Essential oils have been explored for their potential to modulate ruminal ammonia production [47,48,49]. In our study, N-NH_3_ concentrations were not significantly affected by EO treatments, likely due to the administered dosages being insufficient to impact the ruminal microbial population. Variations in N-NH_3_ levels across collection times reflect dynamic microbial activity and feed degradation patterns, suggesting the need for further studies with more frequent sampling intervals to comprehensively understand EO effects on ruminal ammonia dynamics.

In contrast to our in vivo findings, other studies [50,51,52] have documented EO-induced changes in SCFA concentrations, influenced by EO type and dosage. These discrepancies highlight the intricate nature of EO effects on ruminal fermentation and emphasize the necessity for detailed, context-specific research to determine the conditions under which EOs can effectively modulate ruminal metabolism without adverse consequences. 

Our findings contribute to the growing body of evidence on the use of essential oils in ruminant nutrition, indicating that while EOs hold promise for modifying certain aspects of digestion and emissions, their effects are highly variable and dependent on numerous factors including type, dosage, and diet composition. Future research should focus on elucidating the precise mechanisms of action, optimizing dosages, and assessing the long-term implications of EO use on both animal productivity and environmental sustainability.

## 5. Conclusions

Our in vivo study investigated the effects of oregano essential oil (OEO) and thyme essential oil (TEO) at a concentration of 3 mL/kg of concentrate on key parameters in Nellore cattle. Despite the inclusion of these EOs, we observed no significant impact on dry matter intake (DMI), methane production, fermentative parameters, or nutrient digestibility within a dietary composition of 70% forage and 30% concentrate. These findings suggest that the dosages used may have been insufficient to elicit notable changes in the studied variables.

The absence of significant effects underscores the need for further research with higher EO concentrations and more varied formulations to fully elucidate their potential benefits. Such studies should aim to determine the optimal dosages required to achieve meaningful improvements in methane mitigation, nutrient utilization, and overall animal productivity. Moreover, future investigations should consider a range of dietary compositions and ruminant species to better understand the broader applicability and mechanistic action of EOs in confined ruminant diets. Comprehensive research efforts are essential to optimize EO use, ensuring both environmental sustainability and enhanced productivity in ruminant agriculture. 

## Figures and Tables

**Table 1:** Chemical composition of experimental diets on dry matter basis (g kg DM^−1^).

Feed	DM	OM	NDF	ADF	EE	CP	TC	NFC
*Z. mays* silage	339	963	499	307	20	132	811	312
Concentrate	882	905	197	51	60	265	580	383
Diet ratio (70:30)	502	946	408	230	32	172	742	334

ADF, acid detergent fiber; CP, crude protein; DM, dry matter; EE, ether extract; NDF, neutral detergent fiber; NFC, non-fiber carbohydrate; OM, organic matter; TC, total carbohydrate.

**Table 2:** Intake and digestibility by cattle supplemented with essential oils.

Variables	Control	OEO	TEO	Mean	SEM	*p*-Value
DMI (kg DM^−1^)	7.89	7.77	7.76	7.81	0.032	0.9683
IBW (% BW)	1.29	1.27	1.29	1.28	0.001	0.9719
IMW (BW^0.75^)	63.96	63.75	63.96	63.73	0.9339	0.9848
DMd (%)	67.11	66.51	64.09	65.90	15.3369	0.8388
NDFd(%)	60.23	59.35	58.30	59.29	4.1096	0.9296
ADFd(%)	60.95	57.95	55.53	58.14	3.5500	0.9309

SEM, standard error of mean; OEO, *T. vulgaris* essential oil; TEO, *O. vulgare* essential oil; DMI, dry matter intake; IBW, dry matter intake in relation to body weight; IMW, dry matter intake per unit of metabolic weight; DMd, dry matter digestibility; NDFd, neutral detergent fiber digestibility; ADFd, acid detergent fiber digestibility.

**Table 3:** Methane emissions by cattle supplemented with essential oils.

Variables	Control	OEO	TEO	Mean	SEM	*p*-Value
g CH_4_ day^−1^	179.34	177.88	172.96	17.73	67.1323	0.9065
kg CH_4_ year^−1^	65.46	64.93	63.13	64.51	8.9604	0.9630
g CH_4_ kg^−1^ DMI	23.01	24.12	23.02	23.38	2.4679	0.8890
g CH_4_ kg^−1^ BW	0.29	0.29	0.29	0.29	0.0002	0.9849
g CH_4_ kg^−1^BW^0.75^	1.45	1.45	1.43	1.44	0.0014	0.9697
CH_4_ YM (%)	9.97	9.88	9.62	9.82	0.2074	0.9066

SEM, standard error of mean; OEO, *T. vulgaris* essential oil; TEO, *O. vulgare* essential oil; DMI, dry matter intake; BW, body weight; YM, percentage ratio between energy lost by methane and gross energy consumed.

**Table 4:** Ruminal parameters by cattle supplemented with essential oils.

Variables	Treatments			Mean	SEM	*p* Value		
	CON	OEO	TEO			Treat	Time	Treat × Time
pH	6.52	6.55	6.53	6.53	0.0407	0.8989	<0.0001	0.9982
NH_3_-N (ml.dl^−1^)	5.01	5.66	4.87	5.18	0.3898	0.3589	<0.0001	0.5474
SCFA (mM) *	128.13	124.92	123.67	125.57	2.6516	0.5009	<0.0001	0.5427
Acetic (%)	63.61	63.44	62.85	63.30	0.4405	0.4711	0.0027	0.8357
Propionic (%)	21.23	20.23	21.29	20.92	0.6595	0.4765	0.0045	0.8932
Isobutyric (%)	1.30	1.29	1.33	1.31	0.0416	0.7848	<0.0001	0.9931
Butyric (%)	10.64	11.78	11.37	11.26	0.5351	0.3595	0.9250	0.7928
Isovaleric (%)	2.31	2.33	2.20	2.28	0.0936	0.6019	0.0002	0.9858
Valeric (%)	0.90	0.93	0.96	0.93	0.0466	0.6189	<0.0001	0.6537
C_2_:C_3_ * ratio	3.03	3.18	3.00	3.07	0.0683	0.2058	0.0060	0.9654

* interaction (treat × time); SEM, standard error of mean; CON, control; OEO, *T. vulgaris* essential oil; TEO, *O. vulgare* essential oil; NH_3_-N, ammoniacal nitrogen; SCFA, short-chain fatty acid; C_2_:C_3,_ acetate to propionate ratio.

## Data Availability

The raw data supporting the conclusions of this article will be made available by the authors on request.
